# Personalizing the Use of a Intermittently Scanned Continuous Glucose Monitoring Device in Individuals With Type 1 Diabetes: A Cost-Effectiveness Perspective in the Netherlands (FLARE-NL 9)

**DOI:** 10.1177/19322968221109841

**Published:** 2022-07-09

**Authors:** Sajad Emamipour, Peter R. van Dijk, Henk J.G. Bilo, Mireille A. Edens, Onno van der Galiën, Maarten J. Postma, Talitha L. Feenstra, Job F. M. van Boven

**Affiliations:** 1 Department of Clinical Pharmacy and Pharmacology, University of Groningen, University Medical Center Groningen, Groningen, The Netherlands; 2 Department of Endocrinology, University of Groningen, University Medical Center Groningen, Groningen, The Netherlands; 3 Diabetes Centre, Isala, Zwolle, The Netherlands; 4 Department of Internal Medicine, University of Groningen, University Medical Center Groningen, Groningen, The Netherlands; 5 Department of Innovation and Science, Isala, Zwolle, The Netherlands; 6 Achmea, Zeist, The Netherlands; 7 Department of Health Sciences, University of Groningen, University Medical Center Groningen, Groningen, The Netherlands; 8 Department of Economics, Econometrics and Finance, Faculty of Economics and Business, University of Groningen, Groningen, The Netherlands; 9 Groningen Research Institute of Pharmacy, Faculty of Science and Engineering, University of Groningen, Groningen, The Netherlands; 10 National Institute for Public Health and the Environment (RIVM), Bilthoven, The Netherlands

**Keywords:** cost-effectiveness analysis, hypoglycemia, intermittently scanned continuous glucose monitoring, type 1 diabetes mellitus

## Abstract

**Aims:**

Intermittently scanned continuous glucose monitoring (isCGM) is a method to monitor glucose concentrations without using a finger prick. Among persons with type 1 diabetes (T1D), isCGM results in improved glycemic control, less disease burden and improved health-related quality of life (HRQoL). However, it is not clear for which subgroups of patients isCGM is cost-effective. We aimed to provide a real-world cost-effectiveness perspective.

**Methods:**

We used clinical data from a 1-year nationwide Dutch prospective observational study (N = 381) and linked these to insurance records. Health-related quality of life was assessed with the EQ-5D-3L questionnaire. Individuals were categorized into 4 subgroups: (1) frequent hypoglycemic events (58%), (2) HbA1c > 70 mmol/mol (8.5%) (19%), (3) occupation that requires avoiding finger pricks and/or hypoglycemia (5%), and (4) multiple indications (18%). Comparing costs and outcomes 12 months before and after isCGM initiation, incremental cost-effectiveness ratios (ICERs) were calculated for the total cohort and each subgroup from a societal perspective (including healthcare and productivity loss costs) at the willingness to pay of €50,000 per quality-adjusted life year (QALY) gained.

**Results:**

From a societal perspective, isCGM was dominant in all subgroups (ie higher HRQoL gain with lower costs) except for subgroup 1. From a healthcare payer perspective, the probabilities of isCGM being cost-effective were 16%, 9%, 30%, 98%, and 65% for the total cohort and subgroup 1, 2, 3, and 4, respectively. Most sensitivity analyses confirmed these findings.

**Conclusions:**

Comparing subgroups of isCGM users allows to prioritize them based on cost-effectiveness. The most cost-effective subgroup was occupation-related indications, followed by multiple indications, high HbA1c and the frequent hypoglycemic events subgroups. However, controlled studies with larger sample size are needed to draw definitive conclusions.

## Introduction

Self-monitoring of blood glucose (SMBG) using finger pricks is a well-established method to monitor blood glucose in persons with type 1 diabetes (T1D). However, SMBG with finger pricks can be bothersome, and its efficacy depends on patients’ monitoring adherence.^1^ It has been reported that around two-thirds of individuals with T1D do not perform daily SMBG.^2^ To overcome these issues, continuous glucose monitoring (CGM) and intermittently scanned continuous glucose monitoring (isCGM) have been developed.

The isCGM is a factory-calibrated sensor that measures glucose concentrations in the interstitial fluid. The FreeStyle Libre™ (FSL, Abbott Diabetes Care, Witney, United Kingdom) is an isCGM system that was introduced in 2014 and is currently widely used in T1D care.^1^ Several randomized clinical trials (RCTs) were conducted to assess the effectiveness of using the isCGM systems in individuals with T1D. In these studies, significant reductions in hypoglycemic events were observed.^3–5^ Subsequent real-world observational studies demonstrated improvements in glycemic control and quality of life, less disease burden and a decrease in the number of hospital admissions and days of work absenteeism.^1,6–11^

While the isCGM has shown its potential to reduce costs of T1D-related morbidities, the device incurs a certain cost. Therefore, cost-effectiveness assessment should be conducted. To date, only a few economic evaluations of the isCGM have been performed. One study estimated an incremental cost-effectiveness ratio (ICER) of €28 000 per quality-adjusted life year (QALY) in a Swedish setting.^12^ Another study estimated that isCGM would result in an ICER of €6195 per reduced risk of hypoglycemic events in Spain.^13^ A recent cost-effectiveness study in China showed that isCGM was dominant compared to SMBG from a Chinese societal perspective for T1D.^14^ However, in these studies, it was unclear which subgroup(s) of users benefit more in terms of quality of life and what the cost-effectiveness in the various subgroups is.

Given the limited number of estimated cost-effectiveness studies and the paucity of data concerning the cost-effectiveness of isCGM in different subgroups of users, further evidence is required to provide recommendations for which subgroups the isCGM is most cost-effective. Using data from a prospective nationwide registry among Dutch isCGM users, using the FSL-1 (the FLARE-NL registry),^1^ we aimed to evaluate the cost-effectiveness of using the isCGM in subgroups of individuals with T1D in a real-world setting.

## Materials and Methods

### Study Design

This is a cost-effectiveness study using observational data to assess the cost-effectiveness of isCGM (FSL, Abbott Diabetes Care, Witney, United Kingdom; the first generation of the device [FSL-1]) in individuals with T1D and an indication for use of isCGM. We determined the cost per QALY gained for the total study group as well as for several subgroups as defined in the Dutch FLARE-NL registry with predefined indications of isCGM users.^1^ A before-after design was used in the register. The study is reported according to the CHEERS guidelines for economics evaluations.^15^

### Study Setting and Population

We have used the data from the prospective Dutch FLARE-NL registry, which included participants from 88 hospitals across the Netherlands. The registry was started in year 2016 with 1-year follow-up (for more details see Supplementary Materials, section “Study setting”). In this study, 4 subgroups were defined: patients with (1) frequent (self-reported) hypoglycemic events (2) high HbA1c levels (>70 mmol/mol, 8.5%), (3) a critical occupation that requires avoiding finger pricks and/or hypoglycemia, and (4) individuals with more than one of these indications, grouped separately as “multiple indications” (for more details see Supplementary Materials, section “Study population”). The main reason for selecting these subgroups was to identify the T1D individuals based on both clinical (subgroups 1 and 2) and nonclinical (subgroup 3) indications. More details on these subgroups have been described previously.^1^

### Outcomes

In the registry, several clinical and patient-reported outcomes were available at baseline, month 6 and month 12 after starting to use the isCGM of which HbA1c (determined using standardized laboratory procedures) was one of the key clinical outcomes. Other outcomes were reported by the individuals including the number of moderate (self-measured glucose levels < 3 mmol/L) to severe (patient in need of third-party help) hypoglycemic episodes in the last 6 months (levels 2 and 3 according to the American Diabetes Association criteria),^16^ number of hospitalizations due to diabetes in the previous year, number of working days lost in the past 6 months and daily functioning in the past 6 months. Health-related quality of life was assessed by the 12-item short-form health survey (SF-12), visual analog scale (VAS), and the 3-level version of the EQ-5D, evaluated at the Dutch tariff.^17^ A complete description of the outcomes has been published previously.^1^

### Cost-Effectiveness Analysis

Cost-effectiveness analyses were performed from a societal perspective (ie including both healthcare costs and costs related to inability to work, that is, productivity loss costs) using a 1-year time horizon. The ICER was calculated by the difference in costs divided by the difference in QALYs, comparing the baseline year with the follow-up year. For capturing the uncertainty around the ICER and for plotting the cost-effectiveness acceptability curve (CEAC), we conducted bootstrapping (with 1000 simulations). The willingness to pay (WTP) in the Netherlands varies from €20 000 to €80 000 per QALY depending on the disease burden.^18^ For T1D, based on the proportional shortfall method, a WTP threshold of €50 000 per QALY is deemed appropriate to establish cost-effectiveness. For more details see Supplementary Materials, section “Costs-effectiveness analysis.”

### Sensitivity Analyses

Several sensitivity analyses were performed from a healthcare payer perspective. The first included the direct healthcare costs only. For this purpose, the productivity losses were excluded. For HRQoL measured by the EQ-5D-3L where the ceiling effects were present, we therefore did the second sensitivity analysis in which we excluded the individuals with perfect HRQoL (ie a score of 1) at baseline. The third sensitivity analysis was performed by including diabetes-related costs only. For this purpose, a group of experts reviewed the cost categories with their descriptions to extract the diabetes-related costs. The fourth sensitivity analysis was performed among individuals who used a finger prick at least 4 times a day. This was done to assess the current Dutch criterion for reimbursement. Finally, a cost-effectiveness analysis was performed using a short-term follow-up period (ie 6 months), which implied a larger sample size (N = 597).

## Results

### Study Population Selection and Characteristics

**Figure 1.**
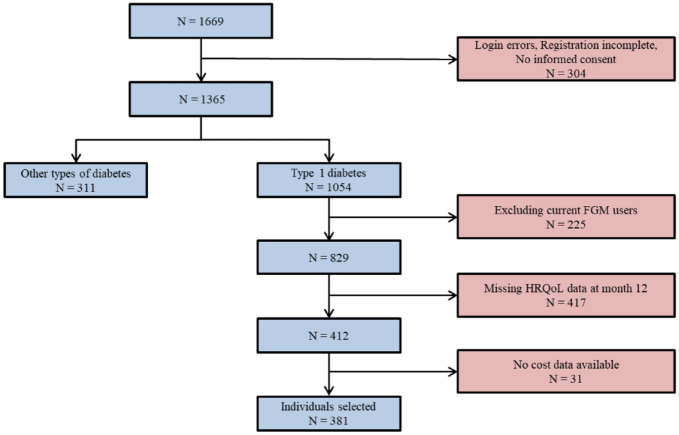
Flowchart of the population selection. Abbreviations: isCGM, intermittently scanned continuous glucose monitoring; HRQoL, health-related quality of life.

Figure 1 shows the flowchart of the population selection process for this study. From the original FLARE-NL registry (N = 1669), 311 had type 2 diabetes (T2D) and 225 were existing isCGM users. Others were excluded due to missing cost or outcome data resulting in a final population of 381 individuals.

Table 1 shows the baseline characteristics of the study population. Among the 4 subgroups, subgroup 1 comprised most (n = 222, 58.3%) of the cohort followed by subgroup 2 (n = 73, 19.2%), subgroup 4 (n = 66, 17.3%), and subgroup 3 (n = 20, 5.2%). Subgroup 3 (critical occupation) had the highest male percentage, EQ-5D-3L, VAS score as well as the youngest individuals. The highest HbA1c level was observed in subgroup 2 (high HbA1c). Individuals with multiple indications (subgroup 4) had the highest number of hypoglycemic events and subgroup 1 (frequent hypoglycemic events) had the highest HRQol score. Cardiovascular problems were more frequently reported by patients in subgroup 4 (multiple indications) and subgroup 3 (critical occupation). Retinopathy and neuropathy were more prevalent in subgroup 3 (critical occupation) and nephropathy reported more in multiple indications (subgroup 4). In all subgroups, most of the individuals (>93.0%) were using insulin for controlling glucose and did not use any other oral antidiabetic medicines. Supplementary Table S4 compares the baseline characteristics of included individuals (N = 381) and those that were excluded (N = 448) due to missing HRQoL at month 12 or missing cost data. The individuals excluded were more often male, had a lower age and higher HbA1c at baseline, with less hypoglycemic episodes. However, their quality of life and costs at baseline was comparable to the individuals included. Supplementary Table S5 shows the change in HbA1c and the number of hypoglycemic events from baseline to month 12. While HbA1c levels decreased in all subgroups, the number of hypoglycemic events increased in subgroup 3.

**Table 1.** Baseline Characteristics of the Study Population (*N* = 381).

Characteristics	Total	Subgroup 1 (frequent hypoglycemic events)	Subgroup 2 (high HbA1c)	Subgroup 3 (critical occupation)	Subgroup 4 (multiple indications)
N, (%)	381 (100.0%)	222 (58.3%)	73 (19.2%)	20 (5.2%)	66 (17.3%)
Male, (%)	193 (50.7%)	115 (51.8%)	30 (41.1%)	17 (85.0%)	31 (47.0%)
Age, (SD)	45.6 (15.9)	46.3 (16.4)	41.7 (14.6)	38.7 (11.0)	49.5 (15.3)
HbA1c mmol/mol, (SD)	61.9 (12.4)	57.1 (8.9)	76.6 (9.7)	62.2 (17.8)	61.7 (10.9)
HbA1c, %, (SD)	7.8 (1.1)	7.4 (0.8)	9.2 (0.9)	7.8 (1.6)	7.8 (1.0)
Hypoglycemic episodes*	45 [20, 92]	50 [23, 100]	25 [11, 50]	30 [15, 60]	55 [25, 100]
Cardiovascular history (%)	50 (13.1%)	26 (11.7%)	8 (11.0%)	4 (20.0%)	12 (18.2%)
Retinopathy (%)	72 (18.9%)	36 (16.2%)	16 (21.9%)	7 (35.0%)	13 (19.7%)
Neuropathy(%)	58 (15.2%)	30 (13.5%)	11 (15.1%)	5 (20.0%)	12 (18.2%)
Nephropathy (%)	37 (9.7%)	19 (8.6%)	5 (6.8%)	2 (10.0%)	11 (16.7%)
Medication					
Insulin monotherapy (%)	361 (94.8%)	208 (93.7%)	69 (94.5%)	20 (100.0%)	64 (97.0%)
Insulin with metformin (%)	17 (4.5%)	11 (5.0%)	4 (5.5%)	0 (0.0%)	2 (3.0%)
Insulin with metformin and gliclazide, glimepiride, tolbutamide, or glipalamide (%)	3 (0.7%)	3 (1.3%)	0 (0.0%)	0 (0.0%)	0 (0.0%)
HRQol, (SD)	0.84 (0.19)	0.85 (0.18)	0.82 (0.20)	0.81 (0.22)	0.83 (0.20)
VAS, (SD)	69.5 (19.4)	69.2 (20.3)	69.6 (17.5)	72.7 (26.2)	69.3 (15.8)

Abbreviations: SD, standard deviation; HRQoL, health-related quality of life (EQ-5D-3L, Dutch tariff); VAS, visual analogue scale.

* The number of hypoglycemic episodes over the past 6 months (median and interquartile).

### Healthcare Costs and Work Productivity Losses

For the total cohort (N = 381), the mean total annual cost was €11 752 at baseline and decreased to €11 567 during the 1-year follow-up (Supplementary Table S6). The cost varied among the subgroups. While subgroup 2 (high HbA1c) had the highest cost at baseline, subgroup 1 (frequent hypoglycemic events) had the highest cost at month 12. Around 80% of the costs were related to 3 segments: “Pharmacy,” “Medical specialty care,” and “Devices” at both baseline and month 12 (Supplementary Figure S1). The median cost of the isCGM device was €1669 (interquartile range: €1391-€1841) annually (Supplementary Figure S2).

Table 2 shows the average working loss over the past year at baseline and month 12. The number of working days lost decreased in all subgroups.

**Table 2.** The Mean Number of Days with Working Loss in the Year Prior to Baseline and Month 12.

Subgroup	Working loss at the year prior to baseline (in days)	Working loss at the year prior to month 12 (in days)
Total cohort, (mean, SD)	13.0 (59.3)	6.7 (38.5)
Subgroup 1, n = 222 (frequent hypoglycemic events), (mean, SD)	10.9 (55.1)	9.0 (48.3)
Subgroup 2, n = 73 (high HbA1c), (mean, SD)	19.4 (72.8)	5.0 (23.4)
Subgroup 3, n = 20 (critical occupation), (mean, SD)	6.5 (17.1)	1.4 (5.8)
Subgroup 4, n = 66 (multiple indications), (mean, SD)	15.0 (64.4)	2.2 (8.4)

Abbreviations: SD, standard deviation.

### Cost-Effectiveness Analysis

**Figure 2.**
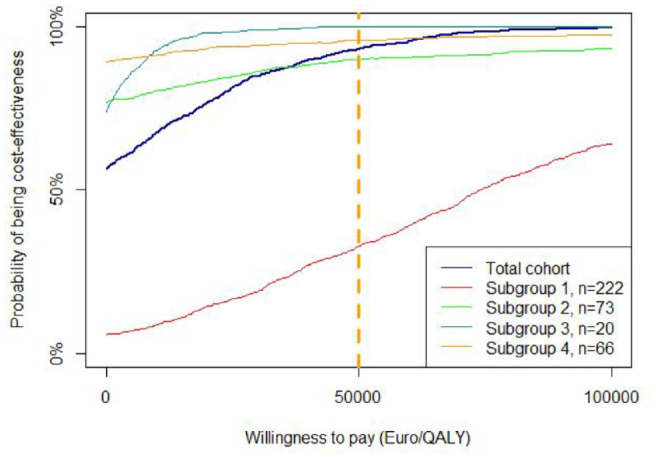
Cost-effectiveness acceptability curves for using the Freestyle Libre in different subgroups with diabetes type 1. Subgroup 1: frequent hypoglycemic events; subgroup 2: high HbA1c; subgroup 3: critical occupation; subgroup 4: multiple indications.

Supplementary Table S6 (the last 2 columns) shows the results of the cost-effectiveness analysis. For the total cohort, the ICER from a societal perspective was dominant (that is, a gain in QALYs was observed, reflecting health benefits, at lower costs). The same was observed in all subgroups except for subgroup 1. For subgroup 1 (frequent hypoglycemic events), the ICER was €72 842 per QALY gained. Supplementary Figure S3 shows the cost-effectiveness clouds for the total cohort as well as for the 4 subgroups. Figure 2 shows the CEAC for the total cohort and the 4 subgroups. The probability of being cost-effective at a threshold of €50,000 per QALY gained, for the total cohort, subgroup 1 (frequent hypoglycemic events), subgroup 2 (high HbA1c), subgroup 3 (critical occupation), and subgroup 4 (multiple indications) was 94%, 32%, 90%, 100%, and 96%, respectively. Since there was no control group in this study, the results should be interpreted as a comparison among different subgroups rather than assessing the cost-effectiveness of the isCGM per se.

### Sensitivity Analyses

There were large differences in ICERs across the different sensitivity analyses as well as the subgroups; however, rankings did not vary so much over the analyses (Table 3). For more details, see Supplementary Materials, section “Sensitivity Analyses.”

**Table 3.** The Ranking of Probability of Each Subgroup Being Cost-Effective Based on Different Sensitivity Analyses.

	Healthcare payer perspective	Excluding score 1 of HRQoL at baseline	Diabetes-related cost segments	Individuals using finger prick at least 4 times a day	Month 6 time point
Subgroup 1, n = 222 (frequent hypoglycemic events)	4	4	4	4	2
Subgroup 2, n = 73 (high HbA1c)	3	3	2	3	3
Subgroup 3, n = 20 (critical occupation)	1	1	1	1	4
Subgroup 4, n = 66 (multiple indications)	2	2	3	2	1

Abbreviations: HRQoL, Health-related quality of life (EQ-5D-3L, Dutch tariff).

Ranking: 1 shows the most favorable and 4 shows the least favorable.

## Discussion

Comparing subgroups in the order of cost-effectiveness showed that individuals with critical occupation had the highest probability of being cost-effective, followed by individuals with multiple indications, high HbA1c, and frequent hypoglycemic events. Since this is a before-after study, findings on absolute rather than relative cost-effectiveness require careful interpretation. These results show that isCGM is cost-effective at a WTP of €50 000 per QALY gained for the subgroups with critical occupation, multiple indications, and high HbA1c, considering the societal perspective. Furthermore, the use of isCGM led to overall cost savings for the total cohort as well as for different subgroups except for the subgroup with frequent hypoglycemic events. From a healthcare payer perspective, the subgroups of individuals with a critical occupation and multiple indications were most cost-effective. The ranking of subgroups in terms of cost-effectiveness was robust over further sensitivity analyses.

In all analyses, individuals using isCGM and having a critical occupation had the most favorable ICER at month 12. The individuals in this subgroup were younger and had lower HRQoL and costs at baseline. This lower starting point and their profession bringing additional problems from either repeated finger pricks or the risk of hypoglycemic events might explain why they benefited the most from using isCGM. However, this subgroup had a less favorable ICER at month 6 compared with month 12. For that reason, and because of the relatively small size of this group, the results for this subgroup should be interpreted carefully. The subgroup of individuals with multiple indications showed stable results and—in all of the different sensitivity analyses—had an ICER lower than €50 000 per QALY gained from a healthcare perspective, while from a societal perspective, isCGM was a dominant strategy. Despite a decrease in hypoglycemic events of 48% for the individuals with frequent hypoglycemic events, HRQoL in this subgroup increased less than the other subgroups (2.6% vs. 3.6% increase in the total cohort), resulting in lower QALY gains. This might imply that a glucose sensor with a hypoglycemic event alarm (such as incorporated in real-time continuous glucose-monitoring devices or newer versions of isGCM) or closed-loop insulin delivery technologies might be a better solution than a device for isGCM without alarms, which was used in the present study. A recent study showed that a closed-loop system was more cost-effective than a CGM plus self-injection of multiple daily insulin for people with T1D in Sweden.^19^

A nationwide prospective observational study in Belgium showed that isCGM led to higher treatment satisfaction (ie was experienced as more convenience compared to SMBG), less work absenteeism and less severe hypoglycemia episodes after 1 year^9^ and as such confirms our findings. The effectiveness of isCGM in terms of decreasing HbA1c and hypoglycemic events has been shown in several clinical trials and observational studies. A recent meta-analysis of clinical trials and observational studies showed that isCGM led to a decrease in HbA1c, especially in individuals with higher HbA1c at baseline^6^ which is in line with our findings. However, they reported an HbA1c decrease of 0.55%, whereas, in our study, a reduction of 0.35% was observed. The smaller decrease in HbA1c level in our study might be due to the longer follow-up time (12 months in our study vs 2-4 months in the review). The largest observational study that assessed the effect of isCGM was performed in the United Kingdom with 7.5 months follow-up.^10^ This study reported a reduction of 5.2 mmol/mol in HbA1c, which is comparable to what we found (3.9 mmol/mol). In both the meta-analysis and the U.K. study, the reduction in HbA1c was reported for isCGM users, and there was no report on SMBG users. In the IMPACT RCT, there was no significant difference in HbA1c reduction between the users of isCGM and SMBG users after 6 months which might be due to the low baseline HbA1c level (6.7%) of the participants.^5^ A reduction of hypoglycemic burden was reported in the IMPACT study, which is in line with the reduction of hypoglycemic events in our study. A descriptive 1-year study assessing the (cost-)effectiveness of isCGM in Spain reported that using isCGM was associated with a 0.39% and 58.9% reduction in HbA1c and hypoglycemic events, respectively.^13^ However, the cost-effectiveness results were not comparable to our study because the authors reported on incremental cost per reduced absolute risk of hypoglycemic events, whereas in our study, an analysis of incremental cost per QALY was performed, that is, the preferred analytic approach in most pharmacoeconomic guidelines. Moreover, the Spanish study had a lower sample size (23) with younger participants. Another study in Sweden reported an ICER of 291 130 Swedish Kroner (€28 736) per QALY gained from a healthcare payer perspective.^12^ This study projected the effect of isCGM using the IQVIA Core Diabetes Model with a lifetime horizon, based on effects taken from the IMPACT trial.^5^ A recent study which also used the IQVIA Core Diabetes Model, showed that isCGM was dominant compared to SMBG in China.^14^ None of these studies estimated subgroup-specific ICERs, as we did in this study. The different time horizon and lower FSL costs could explain the more favorable ICER found in the Swedish study compared with our study.

A strength of this study was the use of a prospective observational data set rather than less generalizable clinical trial data from a more controlled settings, thus our data reflects the real-world situation. Moreover, all cost data were objectively measured using linked claims data. Another strength is that the inclusion criteria for including the participants were not only based on clinical indications (eg HbA1c), but also on the societal aspects (such as having a critical occupation).

There are also several limitations to note for this study. First, there was no control group in this study to compare the isCGM with SMBG. Still the differences in cost-effectiveness between subgroups were deemed to be informative to further tailor the use of isCGM in daily practice.

The observational setting in routine care implied that some characteristics such as body mass index and cholesterol were not available; however, other characteristics such as microvascular and macrovascular and type of treatment were reported. To achieve reliable results, the HRQoL and costs of individuals were compared using a 12 months’ time frame before and after using isCGM and excluding the current isCGM users. Another limitation was that more than half of the study participants did not complete questionnaires at the 1-year follow-up, and hence this study was performed on n = 381 out of the n = 829 that would qualify as new users with T1D in the original FLARE-registry. The reason for this was that filling out the questionnaires was voluntary.^1^ Because of a large percentage of missing values on the outcomes (HRQoL and costs), we did not perform imputation. The baseline characteristics of excluded individuals (age, sex, HbA1c, and hypoglycemic events) were different from the included ones; however, there were no differences in HRQoL, VAS, and costs at baseline which implies that selection bias might have a limited effect on the results. We performed a sensitivity analysis including 6-month follow-up data only, allowing to use of a larger sample of n = 597. This mostly confirmed our findings regarding the ranking of subgroups and resulted in a more favorable ICER for all subgroups except for the subgroup of individuals with a critical occupation.

In addition, HRQoL was measured by the 3-level EQ-5D which is prone to ceiling effects. We handled this limitation in a sensitivity analysis by excluding the individuals with a score of 1 on EQ-5D at baseline. Moreover, merging indications “hypoglycemic unawareness” and “unexpected hypoglycemic” into subgroup 1 (frequent hypoglycemic events) and “sensation loss of fingers” and “endangering others in the case of hypoglycemic events” into subgroup 3 (critical occupation), made these subgroup heterogeneous. The reasons for the merging were to increase the sample size in these subgroups and that having less subgroups would simplify implementation. Finally, a time horizon of 12 months was relatively long for an observational study, but short in relation to the disease duration of diabetes as well as to capture the long-term effects, especially on hypoglycemic events. Using a longer time horizon would imply that also effects in reduction of diabetes complications could be taken into account which would make the ICERs more favorable. Although the short-term effect of isCGM on resource use related to hypoglycemic events was reflected in our claims data, studying the long-term cost-effectiveness of isCGM technology, including its impact on diabetic complications requires a health economic model, which is recommended for future studies.

## Conclusions

From a cost-effectiveness perspective, isCGM seems most favorable for the subgroups with critical occupation, multiple indications, and high HbA1c, respectively. The subgroup of individuals with frequent hypoglycemic events showed a low probability of being cost-effective in most of the analyses. It can be hypothesized that for these persons a real-time glucose sensor with automated preset alarms is more suitable. Results should be interpreted with care since they were based on a before/after comparison of a modest sample size. More evidence, preferably based on controlled studies with longer follow-ups and a larger sample size is needed to determine the cost-effectiveness among subgroups.

## Supplemental Material

sj-docx-1-dst-10.1177_19322968221109841 – Supplemental material for Personalizing the Use of a Intermittently Scanned Continuous Glucose Monitoring (isCGM) Device in Individuals With Type 1 Diabetes: A Cost-Effectiveness Perspective in the Netherlands (FLARE-NL 9) Supplemental material, sj-docx-1-dst-10.1177_19322968221109841 for Personalizing the Use of a Intermittently Scanned Continuous Glucose Monitoring (isCGM) Device in Individuals With Type 1 Diabetes: A Cost-Effectiveness Perspective in the Netherlands (FLARE-NL 9) by Sajad Emamipour, Peter R. van Dijk, Henk J.G. Bilo, Mireille A. Edens, Onno van der Galiën, Maarten J. Postma, Talitha L. Feenstra and Job F. M. van Boven in Journal of Diabetes Science and Technology
