# Supplementary material for: Personalizing the Use of a Intermittently Scanned Continuous Glucose Monitoring Device in Individuals With Type 1 Diabetes: A Cost-Effectiveness Perspective in the Netherlands (FLARE-NL 9)
Source: J Diabetes Sci Technol. 2022 Jul 9;18(1):135–42. doi: 10.1177/19322968221109841 (PMC10899850; doi:10.1177/19322968221109841)
Supplement: sj-docx-1-dst-10.1177_19322968221109841 – Supplemental material for Personalizing the Use of a Intermittently Scanned Continuous Glucose Monitoring (isCGM) Device in Individuals With Type 1 Diabetes: A Cost-Effectiveness Perspective in the Netherlands (FLARE-NL 9) [file sj-docx-1-dst-10.1177_19322968221109841.docx]

**Supplementary materials**

**Materials and Methods**

*Study setting*

Details concerning the prospective Dutch FLARE-NL registry have been published previously [1]. In brief, participants from 88 hospitals across the Netherlands were included in the registry. This registry started with 1186 participants with T1D (N=1669 in total) in 2016 and had a follow-up of one year. The goal of this registry was to assess the real-world effectiveness of the intermittently scanned continuous glucose monitoring (isCGM) with a focus on change in HbA1c levels, number and severity of hypoglycemic events and health-related quality of life (HRQoL) [1]. The isCGM used was the first generation of the FreeStyle Libre™ (FSL, Abbott Diabetes Care, Witney, United Kingdom), which did not have a hypoglycemic event alarm. In addition, all health care use of study participants was obtained from a large Dutch healthcare insurance company (Zilveren Kruis) by linkage, after participants gave their informed consent for this linkage. In order to protect the participants’ privacy, the individuals were pseudonymized by a third party (ZorgTTP.nl) and linked based on sex and date of birth.

*Study population*

In the FLARE-NL registry, both new and existing isCGM users were included. Individuals were categorized according to seven pre-defined indications for isCGM use (see supplementary table S1). The aim of categorizing individuals into these indications was to assess the diabetes population(s) that might benefit most from using the isCGM. A detailed description of these indications and the characteristics of the cohort have been published previously [1]. All participants who were not yet using isCGM, received a isCGM reimbursement of 50% of the actual costs from their health insurer after starting in the registry.

In the current study, we focused on newly initiated users of the isCGM. Therefore, to be able to assess pre and post effects on costs and effects, existing isCGM users were excluded. Additionally, some original indications were merged in order to increase the sample size of each group. Finally, the following four subgroups were applied: patients with (1) frequent hypoglycemic events, (2) high HbA1c levels (> 70 mmol/mol), (3) a critical occupation that requires avoiding finger pricks and/or hypoglycemia, and (4) individuals with more than one of these indications, grouped separately in a “multiple indications” subgroup. Subgroup 3 (critical occupation) included the individuals for whom the loss of sensation due to frequent use of home blood glucose meter (HBGM), can lead to disability and reduced work performance, for instance musicians. It also included persons with occupations for which hypoglycemia events are very dangerous, such as bus and lorry drivers, school teachers, and sports trainers. With hypoglycemia events, they can endanger themselves and/or others. Supplementary table S1 shows how the subgroups relate to the original subgroups in the FLARE-NL registry.

*Costs-effectiveness analysis*

Costs or health care resource use were not measured in the FLARE-NL study. Therefore, healthcare spending was derived from linked health insurance data. Baseline spending referred to spending one year prior to isCGM initiation and follow-up comprised the year after isCGM initiation. Spending included all claims to the mandatory package of health insurance as well as the additional insurance. Claims were categorized into 11 broad categories of health care spending (Table S2), i.e. reimbursement of the isCGM device, pharmacy care, hospital care, home care, mental healthcare, general practitioner care, dentistry, devices, paramedical treatments, abroad/International care and a rest category of other costs, all at 2016 price levels. For the main cost-effectiveness analysis, all these categories were included, and given the limited extent of co-payments in the Dutch health system, it was assumed that these reimbursements would approximate patients’ total health care costs.

In the FLARE-NL study, half of FSL costs was reimbursed [1]. For that reason, in the current study, claimed costs related to FSL were multiplied by two. Moreover, the work absence was measured by a questionnaire covering the past six months, administered at baseline, 6 and 12 months. Since we calculated the productivity loss for the whole year, baseline values were multiplied by 2, while month 6 and month 12 values were added to result in pre-FSL and FSL productivity values. The hourly wage rate was valued at €34.75 per hour (2016), in line with Dutch pharmacoeconomic guidelines [2]. The productivity loss cost was calculated by multiplying the work absence hours with the hourly wage rate. Both costs and the average hourly wage rate were inflated to price levels of the year 2020. The inflation rate for the consumer price and the wage rate are presented in Table S3.

**Results**

*Study population selection and characteristics*

Table S4 compares the baseline characteristics of included individuals (N=381) and those that were excluded (N=448) due to missing HRQoL at month 12 or missing cost data. The individuals excluded were more often male, had a lower age and higher HbA1c at baseline, with less hypoglycemic episodes. However, their quality of life and costs at baseline was comparable to the individuals included. Table S5 shows the change in HbA1c and the number of hypoglycemic events from baseline to month 12. While HbA1c levels decreased in all subgroups, the number of hypoglycemic events increased in subgroup 3.

*Sensitivity analyses*

Table S6 shows the results for the base case analysis (societal perspective). Tables S7-S12 show the ICERs for different analyses. Figures S5-S9 show the probability of being cost-effective for the total cohort and the subgroups based on the sensitivity analyses. The probability of being cost-effective for subgroup 4 (multiple indications) was higher than 50% for all of the sensitivity analyses. Table 3 shows the ranking of the probability of being cost-effective for the subgroups based on each analysis. Like in the main analysis, subgroup 3 (critical occupations) and subgroup 1 (frequent hypoglycemic events) had the highest and the lowest ranking robustly for all sensitivity analyses, respectively.

Table S1: The relation between the indications used in the FLARE-NL registry and the current study

| FLARE-NL registry |  | Current study |
| --- | --- | --- |
| Indication | Description | Subgroup |
| 1 | Having hypoglycemia unawareness and moderate to severe hypoglycemic episodes in the past six months prior to use isCGM | 1 |
| 2 | Having unexpected hypoglycemia despite receiving intensive care in the past six months prior to use isCGM | 1 |
| 3 | Having a mean of HbA1c of higher than 70 mmol/mol (8.5%) over the past one year prior to use isCGM | 2 |
| 4 | Having an occupation that sensation loss of fingers can cause disability (such as musicians) | 3 |
| 5 | Having an occupation that occurring hypoglycemic events can endanger the person him/herself or others (such as bus drivers or school teachers) | 3 |
| 6 | Being eligible for reimbursement based on Dutch regulations in 2016 | Excluded |
| 7 | Individuals who are already users of isCGM on their own costs | Excluded |

The individuals with multiple indications were grouped separately.

Table S2: The healthcare cost segments and their explanations

| **Segment** | **Explanation** |
| --- | --- |
| Abroad/International care | The healthcare cost of individuals outside the Netherlands |
| isCGM | The cost of the isCGM device including the cost of device and the sensor |
| Pharmacy | The cost of outpatient medicines |
| Specialist care | Inpatient and outpatient hospital care |
| Home care | Receiving nursing care at home |
| Mental healthcare | Receiving specialist treatment for mental problems |
| GP | General practitioner care |
| Dentist | Receiving treatment related to dental problems |
| Devices | Costs for devices |
| Paramedical treatments | Receiving paramedic help (physiotherapist; dietician; etc) |
| Other | The healthcare costs that are not in the above segments |

isCGM: Intermittently scanned continuous glucose monitoring; GP: General practitioner

Table S3: The inflation rate for the CPI and wage

| **Year** | **CPI (Year-on-year % change)** | **Wage rate (Year-on-year % change)** | **Source** |
| --- | --- | --- | --- |
| 2015 | 0.6 | 1.4 | Centraal Bureau voor de Statistiek ([Link](https://www.cbs.nl/en-gb/news/2021/02/inflation-rate-1-3-percent-in-2020)) |
| 2016 | 0.3 | 1.8 | Centraal Bureau voor de Statistiek ([Link](https://www.cbs.nl/en-gb/news/2021/02/inflation-rate-1-3-percent-in-2020)) |
| 2017 | 1.4 | 1.4 | Centraal Bureau voor de Statistiek ([Link](https://www.cbs.nl/en-gb/news/2021/02/inflation-rate-1-3-percent-in-2020)) |
| 2018 | 1.7 | 2.0 | Centraal Bureau voor de Statistiek ([Link](https://www.cbs.nl/en-gb/news/2021/02/inflation-rate-1-3-percent-in-2020)) |
| 2019 | 2.6 | 2.5 | Centraal Bureau voor de Statistiek ([Link](https://www.cbs.nl/en-gb/news/2021/02/inflation-rate-1-3-percent-in-2020)) |
| 2020 | 1.3 | 3.0 | Centraal Bureau voor de Statistiek ([Link](https://www.cbs.nl/en-gb/news/2021/02/inflation-rate-1-3-percent-in-2020)) |

CPI: Consumer price index

Table S4: Comparison of the baseline characteristics of the selected individuals with excluded individuals due to lack of follow-up data on HRQoL at month 12

| **Characteristics** | **Individual selected (N=381)** | **Individual excluded (N=448)** | **P-value** |
| --- | --- | --- | --- |
| Male, (%) | 193 (50.7%) | 262 (58.5%) | 0.03 |
| Age, (SD) | 45.6 (15.9) | 40.9 (15.7) | < 0.01 |
| HbA1c mmol/mol, (SD) | 61.9 (12.4) | 65.7 (14.6) | < 0.01 |
| Hypoglycemic events*, n (SD) | 65.4 (73.3) | 52.1 (64.4) | 0.01 |
| HRQoL, (SD) | 0.84 (0.19) | 0.84 (0.20) | 0.98 |
| VAS, (SD) | 69.5 (19.4) | 69.8 (20.4) | 0.84 |
| Costs per patient per year (€) | 7679 (8332) | 7729 (8098) | 0.94 |

*The number of hypoglycemic episodes over the past 6 months

HRQoL: Health-related quality of life (EQ5D); VAS: Visual analogue scale

Table S5: Change in HbA1c and the number of hypoglycemic events at baseline and month 12

|  | **Baseline** | **Month 12** | **Change** |
| --- | --- | --- | --- |
| **HbA1c (mmol/mol) (SD)** |  |  |  |
| *Total cohort, n=381* | *61.9 (12.4)* | *58.0 (10.4)* | *-6.3* |
| Subgroup 1, n=222 (Frequent hypoglycemic events) | 57.1 (8.9) | 55.4 (7.8) | -3.0 |
| Subgroup 2, n=73 (High HbA1c) | 76.6 (9.7) | 67.7 (13.5) | -11.6 |
| Subgroup 3, n=20 (Critical occupation) | 62.2 (17.8) | 59.4 (12.7) | -4.5 |
| Subgroup 4, n=66 (Multiple indications) | 62.0 (10.9) | 56.7 (7.8) | -8.5 |
| **HbA1c (%) (SD)** |  |  |  |
| *Total cohort, n=381* | *7.81% (1.13)* | *7.46% (0.95)* | *-0.35%* |
| Subgroup 1, n=222 (Frequent hypoglycemic events) | 7.38% (0.81) | 7.22% (0.72) | -0.16% |
| Subgroup 2, n=73 (High HbA1c) | 9.16% (0.89) | 8.34% (1.23) | -0.82% |
| Subgroup 3, n=20 (Critical occupation) | 7.84% (1.63) | 7.59% (1.17) | -0.25% |
| Subgroup 4, n=66 (Multiple indications) | 7.82% (1.00) | 7.34% (0.71) | -0.48% |
| **Hypoglycemic events*** |  |  |  |
| *Total cohort, n=381* | *45 [20, 92]* | *26 [12, 80]* | *-19* |
| Subgroup 1, n=222 (Frequent hypoglycemic events) | 50 [23, 100] | 26 [12, 81] | -24 |
| Subgroup 2, n=73 (High HbA1c) | 25 [11, 50] | 25 [12, 50] | 0 |
| Subgroup 3, n=20 (Critical occupation) | 30 [15, 60] | 35 [15, 50] | 5 |
| Subgroup 4, n=66 (Multiple indications) | 55 [25, 100] | 30 [12, 100] | -25 |

*The number of hypoglycemic episodes over the past 6 months (Median and interquartile)

SD: Standard deviation

Figure S1: The breakdown of costs at baseline and month 12 for each subgroup


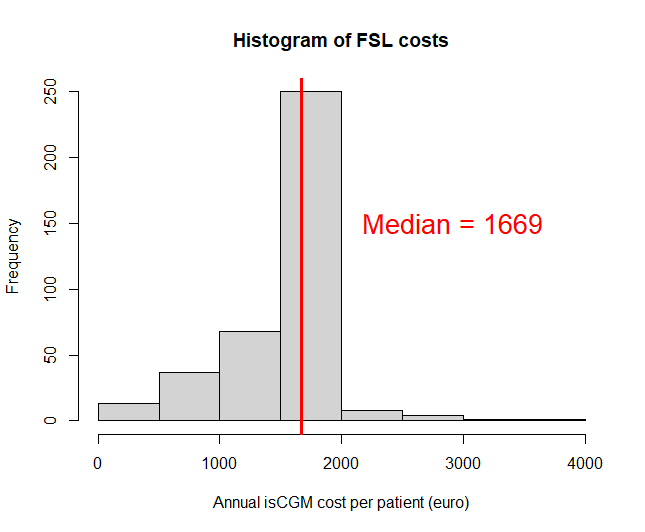


Figure S2: The histogram of annual intermittently scanned continuous glucose monitoring (isCGM) cost per individual


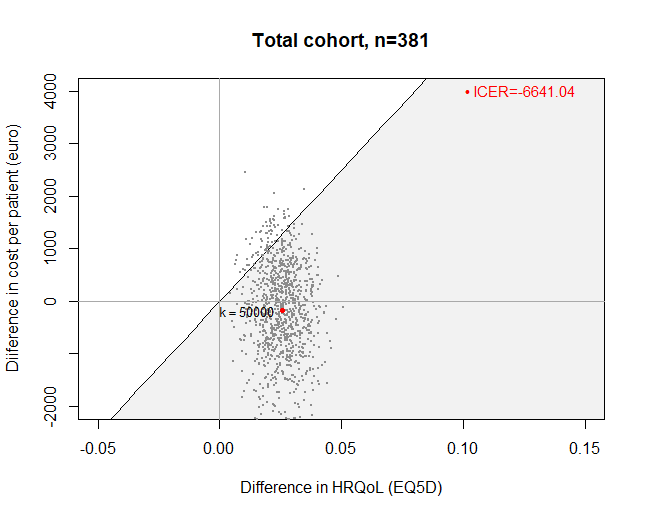


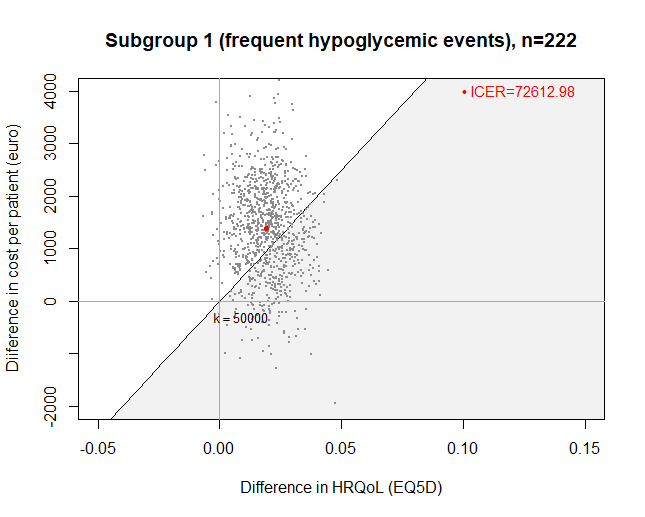

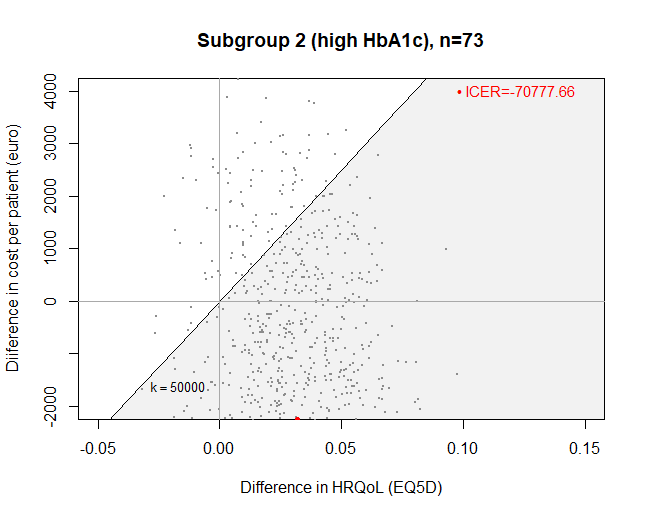

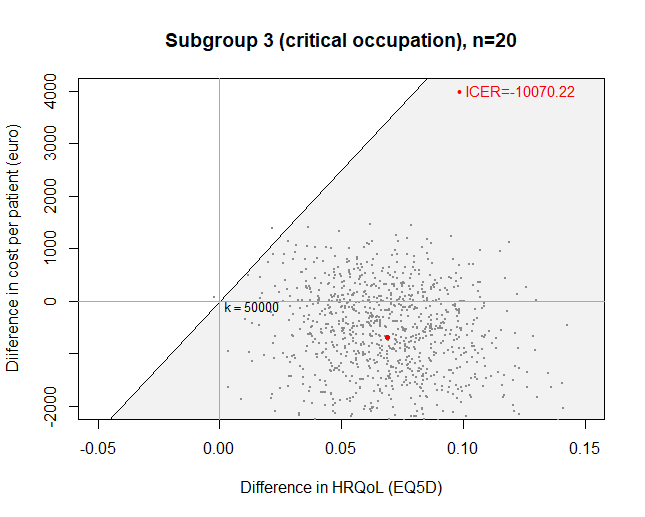

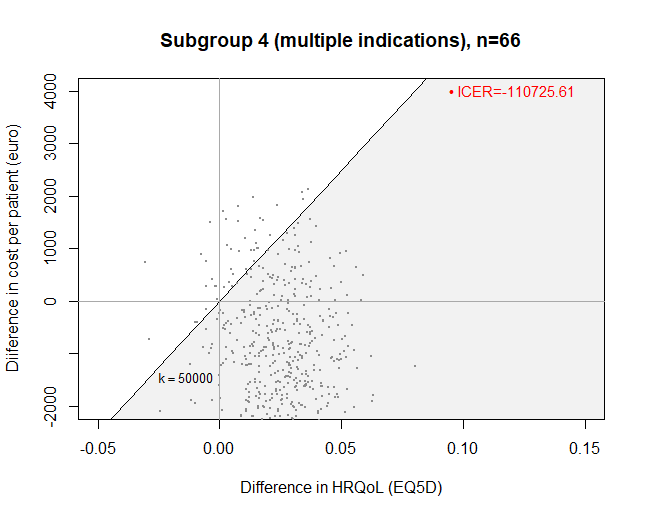


Figure S3: The cost-effectiveness plane for the total cohort and the four subgroups

Table S6: Mean cost, HRQoL and ICER before and after intermittently scanned continuous glucose monitoring (isCGM) initiation in individuals with type 1 diabetes from a societal perspective

|  | **Baseline** | **Month 12** | **ICER (Euro per QALY)** | **Probability of being cost-effective** |
| --- | --- | --- | --- | --- |
| **Total cohort, n=381** |  |  | Dominant | 0.94 |
| HRQoL | 0.84 (0.82, 0.86) | 0.87 (0.85, 0.88) |  |  |
| Cost | €11752 (€9847, €13640) | €11567 (€10141, €13028) |  |  |
| **Subgroup 1, n=222 (frequent hypoglycemic events)** |  |  | 72,613 | 0.32 |
| HRQoL | 0.85 (0.83, 0.87) | 0.87 (0.85, 0.89) |  |  |
| Cost | €10966 (€8894, €13058) | €12362 (€10226, €14633) |  |  |
| **Subgroup 2, n=73 (high HbA1c)** |  |  | Dominant | 0.90 |
| HRQoL | 0.83 (0.79, 0.86) | 0.86 (0.82, 0.9) |  |  |
| Cost | €13807 (€8695, €20072) | €11560 (€9160, €14511) |  |  |
| **Subgroup 3, n=20 (critical occupation)** |  |  | Dominant | 1.00 |
| HRQoL | 0.81 (0.73, 0.89) | 0.88 (0.80, 0.95) |  |  |
| Cost | €7768 (€5123, €10445) | €7085 (€5488, €8882) |  |  |
| **Subgroup 4, n=66 (multiple indications)** |  |  | Dominant | 0.96 |
| HRQoL | 0.83 (0.79, 0.86) | 0.85 (0.81, 0.89) |  |  |
| Cost | €13316 (€8899, €18884) | €10260 (€8883, €11981) |  |  |

Parentheses represent 95% confidence interval; HRQoL: Health-related quality of life (EQ5D); ICER: Incremental cost-effectiveness ratio

Table S7: Mean cost, HRQoL and ICER before and after intermittently scanned continuous glucose monitoring (isCGM) initiation in individuals with type 1 diabetes at month 12 – a healthcare payer perspective (N = 381)

|  | **Baseline** | **Month 12** | **ICER (Euro per QALY)** | **Probability of being cost-effective** |
| --- | --- | --- | --- | --- |
| **Total cohort, n=381** |  |  | 71,363 | 0.16 |
| HRQoL | 0.84 (0.82, 0.86) | 0.87 (0.85, 0.88) |  |  |
| Cost | €7655 (€6955, €8375) | €9481 (€8607, €10472) |  |  |
| **Subgroup 1, n=222 (frequent hypoglycemic events)** |  |  | 106,132 | 0.09 |
| HRQoL | 0.85 (0.83, 0.87) | 0.87 (0.85, 0.89) |  |  |
| Cost | €7565 (€6779, €8339) | €9548 (€8397, €11018) |  |  |
| **Subgroup 2, n=73 (high HbA1c)** |  |  | 70,969 | 0.30 |
| HRQoL | 0.83 (0.78, 0.86) | 0.86 (0.82, 0.89) |  |  |
| Cost | €7690 (€5814, €10011) | €10,020 (€8075, €12226) |  |  |
| **Subgroup 3, n=20 (critical occupation)** |  |  | 13,047 | 0.98 |
| HRQoL | 0.81 (0.73, 0.88) | 0.88 (0.79, 0.95) |  |  |
| Cost | €5731 (€4038, €7685) | €6648 (€5204, €8358) |  |  |
| **Subgroup 4, n=66 (multiple indications)** |  |  | 37,165 | 0.65 |
| HRQoL | 0.83 (0.78, 0.87) | 0.85 (0.81, 0.89) |  |  |
| Cost | €8509 (€6965, €10199) | €9523 (€8162, €11103) |  |  |

Parentheses represent 95% confidence interval; HRQoL: Health-related quality of life (EQ5D); ICER: Incremental cost-effectiveness ratio

Table S8: Mean cost, HRQoL and ICER before and after intermittently scanned continuous glucose monitoring (isCGM) initiation in individuals with type 1 diabetes at month 12 – excluding score 1 of HRQoL at baseline with all cost segments (N = 233) with healthcare costs

|  | **Baseline** | **Month 12** | **ICER (Euro per QALY)** | **Probability of being cost-effective** |
| --- | --- | --- | --- | --- |
| **Total cohort, n=233** |  |  | 28,080 | 0.94 |
| HRQoL | 0.74 (0.72, 0.76) | 0.8 (0.78, 0.82) |  |  |
| Cost | €9127 (€8118, €10205) | €10831 (€9591, €12219) |  |  |
| **Subgroup 1, n=133 (frequent hypoglycemic events)** |  |  | 39,111 | 0.67 |
| HRQoL | 0.75 (0.73, 0.78) | 0.81 (0.78, 0.83) |  |  |
| Cost | €9009 (€7803, €10320) | €11060 (€9274, €13225) |  |  |
| **Subgroup 2, n=47 (high HbA1c)** |  |  | 31,134 | 0.75 |
| HRQoL | 0.73 (0.68, 0.77) | 0.79 (0.74, 0.85) |  |  |
| Cost | €9608 (€6936, €13055) | €11680 (€8824, €14840) |  |  |
| **Subgroup 3, n=12 (critical occupation)** |  |  | 4920 | 1.0 |
| HRQoL | 0.68 (0.58, 0.77) | 0.81 (0.69, 0.91) |  |  |
| Cost | €6606 (€4166, €9575) | €7222 (€5267, €9748) |  |  |
| **Subgroup 4, n=41 (multiple indications)** |  |  | 7602 | 0.98 |
| HRQoL | 0.72 (0.67, 0.76) | 0.78 (0.72, 0.84) |  |  |
| Cost | €9689 (€7332, €12172) | €10157 (€8221, €12547) |  |  |

Parentheses represent 95% confidence interval; HRQoL: Health-related quality of life (EQ5D); ICER: Incremental cost-effectiveness ratio

Table S9: Diabetes-related cost segments

| **Segment** | **Note** |
| --- | --- |
| isCGM | Sensor and reader |
| Specialist care | Diabetes-related care |
| General practitioner visits | Visits, e-mail and phone consultations |
| Devices | Lancets and test materials |
| Paramedical treatments | Diet and foot care |
| Pharmacy | Insulin and Glucagon |

isCGM: Intermittently scanned continuous glucose monitoring (isCGM)

Figure S4: The breakdown of diabetes-related costs at baseline and month 12 for each subgroup

Table S10: Mean cost, HRQoL and ICER before and after isCGM initiation in individuals with type 1 diabetes at month 12 – including just diabetes-related cost segments (N = 381) with healthcare costs

|  | **Baseline** | **Month 12** | **ICER (Euro per QALY)** | **Probability of being cost-effective** |
| --- | --- | --- | --- | --- |
| **Total cohort, n=381** |  |  | 47,879 | 0.55 |
| HRQoL | 0.84 (0.82, 0.86) | 0.87 (0.85, 0.88) |  |  |
| Cost | €3974 (€3772, €4173) | €5195 (€4990, €5397) |  |  |
| **Subgroup 1, n=222 (frequent hypoglycemic events)** |  |  | 62,006 | 0.30 |
| HRQoL | 0.85 (0.83, 0.87) | 0.87 (0.85, 0.89) |  |  |
| Cost | €3943 (€3699, €4211) | €5131 (€4890, €5389) |  |  |
| **Subgroup 2, n=73 (high HbA1c)** |  |  | 43,383 | 0.59 |
| HRQoL | 0.82 (0.79, 0.87) | 0.86 (0.81, 0.9) |  |  |
| Cost | €4274 (€3736, €4854) | €5638 (€5094, €6220) |  |  |
| **Subgroup 3, n=20 (critical occupation)** |  |  | 17,108 | 0.98 |
| HRQoL | 0.81 (0.73, 0.88) | 0.88 (0.8, 0.95) |  |  |
| Cost | €2625 (€2106, €3160) | €3810 (€3335, €4349) |  |  |
| **Subgroup 4, n=66 (multiple indications)** |  |  | 43,501 | 0.59 |
| HRQoL | 0.83 (0.78, 0.86) | 0.85 (0.81, 0.89) |  |  |
| Cost | €4157 (€3679, €4701) | €5341 (€4889, €5801) |  |  |

Parentheses represent 95% confidence interval; HRQoL: Health-related quality of life (EQ5D); ICER: Incremental cost-effectiveness ratio

Table S11: Mean cost, HRQoL and ICER before and after isCGM initiation in individuals with type 1 diabetes at month 12 – individuals using finger-prick at least 4 times a day (N=314) with healthcare costs

|  | **Baseline** | **Month 12** | **ICER (Euro per QALY)** | **Probability of being cost-effective** |
| --- | --- | --- | --- | --- |
| **Total cohort, n=314** |  |  | 71,620 | 0.16 |
| HRQoL | 0.84 (0.83, 0.86) | 0.87 (0.86, 0.89) |  |  |
| Cost | €7544 (€6893, €8190) | €9649 (€8706, €10769) |  |  |
| **Subgroup 1, n=185 (frequent hypoglycemic events)** |  |  | 99,138 | 0.12 |
| HRQoL | 0.86 (0.83, 0.88) | 0.88 (0.86, 0.9) |  |  |
| Cost | €7594 (€6690, €8604) | €9821 (€8437, €11478) |  |  |
| **Subgroup 2, n=57 (high HbA1c)** |  |  | 69,365 | 0.33 |
| HRQoL | 0.81 (0.76, 0.86) | 0.85 (0.81, 0.89) |  |  |
| Cost | €7102 (€5769, €8526) | €9777 (€7821, €12045) |  |  |
| **Subgroup 3, n=15 (critical occupation)** |  |  | 13,980 | 0.96 |
| HRQoL | 0.81 (0.72, 0.89) | 0.88 (0.79, 0.95) |  |  |
| Cost | €6146 (€3960, €8941) | €7127 (€5338, €9370) |  |  |
| **Subgroup 4, n=57 (multiple indications)** |  |  | 44,814 | 0.58 |
| HRQoL | 0.85 (0.81, 0.89) | 0.88 (0.85, 0.92) |  |  |
| Cost | €8196 (€6791, €9739) | €9631 (€8139, €11425) |  |  |

Parentheses represent 95% confidence interval; HRQoL: Health-related quality of life (EQ5D); ICER: Incremental cost-effectiveness ratio

Table S12: Mean cost, HRQoL and ICER before and after isCGM initiation in individuals with type 1 diabetes – at month six time point (N=597) with healthcare costs

|  | **Baseline** | **Month 6** | **ICER (Euro per QALY)** | **Probability of being cost-effective** |
| --- | --- | --- | --- | --- |
| **Total cohort, n=597** |  |  | 33,719 | 0.90 |
| HRQoL | 0.84 (0.83, 0.85) | 0.87 (0.86, 0.88) |  |  |
| Cost | €3779 (€3496, €4054) | €4667 (€4359, €4981) |  |  |
| **Subgroup 1, n=330 (Frequent hypoglycemic events)** |  |  | 27,464 | 0.91 |
| HRQoL | 0.85 (0.84, 0.87) | 0.88 (0.86, 0.89) |  |  |
| Cost | €3777 (€3410, €4140) | €4518 (€4154, €4904) |  |  |
| **Subgroup 2, n=133 (High HbA1c)** |  |  | 57,458 | 0.39 |
| HRQoL | 0.83 (0.8, 0.86) | 0.85 (0.82, 0.88) |  |  |
| Cost | €3567 (€3067, €4134) | €4920 (€4217, €5722) |  |  |
| **Subgroup 3, n=38 (Critical occupation)** |  |  | 224,105 | 0.24 |
| HRQoL | 0.85 (0.81, 0.9) | 0.86 (0.8, 0.91) |  |  |
| Cost | €3079 (€2391, €3820) | €4382 (€3408, €5607) |  |  |
| **Subgroup 4, n=96 (Multiple indications)** |  |  | 16,098 | 0.92 |
| HRQoL | 0.82 (0.79, 0.86) | 0.86 (0.82, 0.88) |  |  |
| Cost | €4365 (€3606, €5264) | €4942 (€4245, €5734) |  |  |

Parentheses represent 95% confidence interval; HRQoL: Health-related quality of life (EQ5D); ICER: Incremental cost-effectiveness ratio

Figure S5: The probability of being cost-effective for the total cohort based on different sensitivity analyses

Figure S6: The probability of being cost-effective for the subgroup 1 (frequent hypoglycemic events) based on different sensitivity analyses

Figure S7: The probability of being cost-effective for the subgroup 2 (high HbA1c) based on different sensitivity analyses

Figure S8: The probability of being cost-effective for the subgroup 3 (critical occupation) based on different sensitivity analyses

Figure S9: The probability of being cost-effective for the subgroup 4 (multiple indications) based on different sensitivity analyses

**Reference**

1. Fokkert, M., et al., *Improved well-being and decreased disease burden after 1-year use of flash glucose monitoring (FLARE-NL4).* BMJ Open Diabetes Research and Care, 2019. **7**(1): p. e000809.

2. *Guideline for economic evaluation, available from:* [*https://english.zorginstituutnederland.nl/publications/reports/2016/06/16/guideline-for-economic-evaluations-in-healthcare*](https://english.zorginstituutnederland.nl/publications/reports/2016/06/16/guideline-for-economic-evaluations-in-healthcare)*.* 2016.
